# Real-time monitoring of glutathione in living cells using genetically encoded FRET-based ratiometric nanosensor

**DOI:** 10.1038/s41598-020-57654-y

**Published:** 2020-01-22

**Authors:** Mohammad Ahmad, Naser A. Anjum, Ambreen Asif, Altaf Ahmad

**Affiliations:** 1 Department of Botany, School of Chemical and Life Sciences, Jamia Hamdard, New Delhi, India; 2 Department of Botany, Faculty of Life Sciences, Aligarh Muslim University, Aligarh, India

**Keywords:** Fluorescent proteins, Abiotic

## Abstract

Reduced glutathione (GSH) level inside the cell is a critical determinant for cell viability. The level of GSH varies across the cells, tissues and environmental conditions. However, our current understanding of physiological and pathological GSH changes at high spatial and temporal resolution is limited due to non-availability of practicable GSH-detection methods. In order to measure GSH at real-time, a ratiometric genetically encoded nanosensor was developed using fluorescent proteins and fluorescence resonance energy transfer (FRET) approach. The construction of the sensor involved the introduction of GSH binding protein (YliB) as a sensory domain between cyan fluorescent protein (CFP; FRET donor) and yellow fluorescent protein (YFP; FRET acceptor). The developed sensor, named as FLIP-G (Fluorescence Indicator Protein for Glutathione) was able to measure the GSH level under *in vitro* and *in vivo* conditions. When the purified FLIP-G was titrated with different concentrations of GSH, the FRET ratio increased with increase in GSH-concentration. The sensor was found to be specific for GSH and also stable to changes in pH. Moreover, in live bacterial cells, the constructed sensor enabled the real-time quantification of cytosolic GSH that is controlled by the oxidative stress level. When expressed in yeast cells, FRET ratio increased with the external supply of GSH to living cells. Therefore, as a valuable tool, the developed FLIP-G can monitor GSH level in living cells and also help in gaining new insights into GSH metabolism.

## Introduction

Reduced glutathione (GSH), a ubiquitous non-protein thiol of antioxidant system, is widely distributed in microbes, plants and animals. The GSH plays crucial roles in cellular defense against xenobiotics and reactive oxygen species^1,2^. During oxidative stress, reduced form of glutathione (GSH) is converted into oxidized form (GSSG)^3^. The GSSG/GSH ratio is used to determine the redox status of the cells. Moreover, the deficiency of GSH has been reported to significantly impact the health of both humans^4^ and plants^2^.

In humans, the level of GSH varies significantly, as plasma and urine has lower GSH level; whereas, it is found in millimolar concentrations at intracellular level^5^. Measurement of blood GSH level is a measure of whole-body GSH, redox status and risk of developing immunomediated disorders^5^. In plant system as well as in humans, the status of the reduced form of cellular GSH and also that of its redox state is indicative of the reduced cellular environment capable of combating oxidative stress-impacts^2,4^. *In vivo* oxidative stress level can also be monitored through the measurement of intracellular GSH concentration of the cells^2,4,6,7^. Techniques such as fluorimetry, bioluminometery, HPLC and LC/MS have so far been used to detect and quantify different forms of GSH in biological samples^8^. However, disruption and fractionation of tissues are carried out for these techniques^9^; therefore, could not determine the metabolite concentration in real-time *in vivo* with high spatio-temporal resolution. In recent years, sensors based on fluorescent dye or quantum dot have been constructed for the measurement of intracellular metabolites^10^. Unfortunately, the toxicity and delivery problems are associated with these kind of sensors and the determination of the analyte in real-time is not possible. Therefore, to understand the real-time dynamics of cellular GSH *in vivo*, a non-invasive visible reporter is needed.

In recent years, green fluorescent protein (GFP) variants and ligand sensing domains have been used to design genetically encoded sensors based on fluorescence resonance energy transfer (FRET). Additionally, the designing of different sensors has used the circularly permutated fluorescent protein (cpFP), where the original N and C termini were replaced by a short peptide linker in order to alter the pKa values and orientation of chromophore from their original counterpart^11,12^. This cpFP has been adapted in designing ATP indicator, ATeam^13^ and NADPH sensor (iNAP)^14^. The conformational change in the sensory domain is involved in the detection of the target metabolite through the genetically encoded FRET-based sensor (a tandemly fused protein). In FRET, dipole-dipole coupling leads to the transfer of energy from an excited donor fluorophore to a neighboring acceptor fluorophore. The occurrence of FRET between two fluorescent moieties is associated with changes in the conformation of sensory domain. Binding of ligand to the sensory domain increases the energy transfer efficiency from donor fluorescent protein to the acceptor fluorescent protein. Finally, ratio of emission intensities of the donor and acceptor fluorescent proteins (FRET ratio) reports dynamics of level of the target metabolite. Successful real-time *in vivo* detection and imaging of calcium, cAMP, malonyl CoA and heme have been performed earlier employing this FRET-based strategy^15–18^. The status of the activity of focal adhesion kinase in the regulation of cell adhesion, migration and proliferation in response to growth factor has been measured using FRET-based biosensor^19^. The efficacy of drugs in patients with chronic myeloid leukemia (CML) has also been measured through FRET-based biosensor-mediated monitoring of the activity of BCR-ABL kinase that is causatively expressed in CML patients^20^.

In this study, GFP derivatives (i.e. CFP and YFP) were exploited to create an improved cellular GSH sensor based on GSH binding protein (YliB). The YliB from *Escherichia coli* serves as a proxy to report the cellular GSH level. A change in FRET-efficiency in bound and unbound states indicates the GSH level that can be measured using the constructed herein GSH sensor named as FLIP-G (Fluorescence Indicator Protein for Glutathione). FLIP-G was able to report the GSH level with real-time changes in fluorescence intensity ratio in a single yeast cell. Thus, FLIP-G could contribute in getting more insights into the cellular and subcellular variations in GSH level that may occur in many pathological and stress conditions, and during normal cellular growth and signaling.

## Results and Discussion

### Structure-guided engineering and designing of FLIP-G

**Figure 1:**
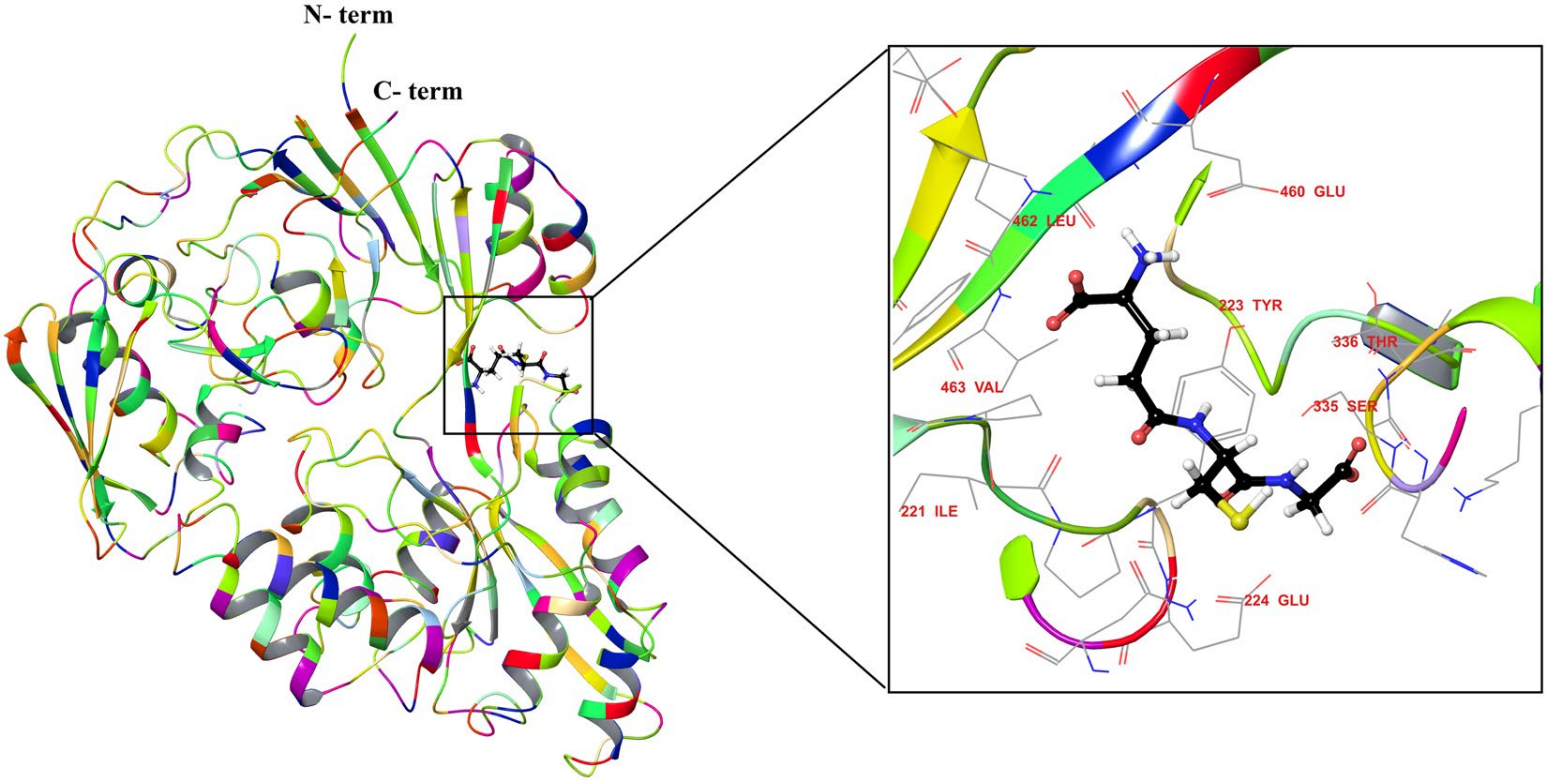
Crystal structure of GSH binding protein in complex with reduced glutathione (GSH). Ligand is shown in ball and stick form. Extended view of active site with ligand shows the interaction with specific amino acids.

Crystal structure of YliB protein with a resolution of 2.72 Å was retrieved from RCSB-PDB. X-ray crystal structure of YliB protein was used for the molecular docking studies of GSH employing glide docking algorithm and dock poses were acquired (Fig. 1). GSH exhibited notable interaction with YliB at the binding cleft. The best docking and glide score obtained herein for GSH confirmed the high affinity of this ligand towards the active site. Additionally, the results of docking studies disclosed the interaction of GSH with YliB through hydrophobic interaction, polar interaction and charged interaction (Fig. S2 in ESI). Binding of GSH causes significant conformational changes between two globular domains of YliB^21^. It was argued that the conformational change in bound and unbound state of YliB would lead to a change in FRET efficiency between the fluorophores. Based on ligand specificity and satisfactory conformational changes, YliB was carefully chosen as a ligand-sensing domain for the development of a nanosensor for monitoring GSH *in vitro and in vivo*.

**Figure 2:**
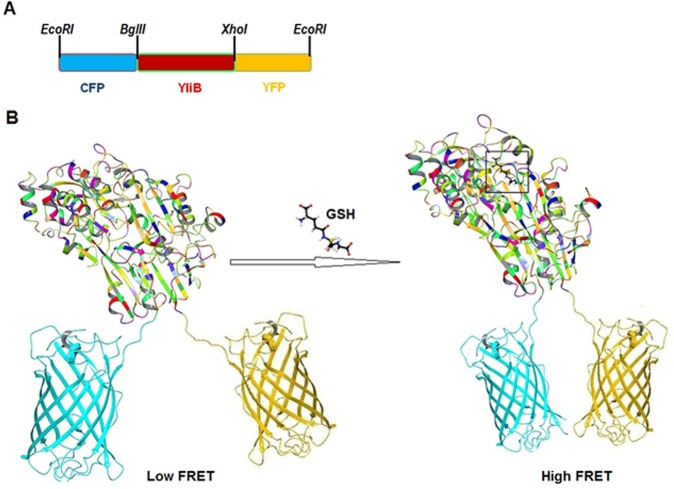
Designing of FRET-based GSH indicator: (**A**) Genetic map of the FLIP-G construct representing the genes and arrangement of restriction sites; (**B**) Energy transfer illustration of the developed sensor. In unbound state (left), open form of FLIP-G causes less energy transfer (i.e. low FRET) by the excitation of CFP at 435 nm. In ligand bound form, a conformational change fetches cyan and yellow FP in close proximity, which increases energy transfer efficiency (i.e. high FRET) from CFP to YFP.

By removing the targeting signal sequence from YliB, nucleotide sequence of this protein was inserted between CFP and YFP gene sequence and cloned in various expression vectors (Fig. 2A). CFP and YFP as a donor and acceptor fluorophores is the most promising pair among the various fluorescent proteins used in designing of FRET nanosensors^22–24^. The proximity changes in both fluorophores in ligand bound and unbound states are presented (Fig. 2B). The GSH binding causes the conformational change in the YliB, fetches close both the FP pair, which is reflected in the form of high FRET-efficiency. Additionally, changes in the concentration of target metabolite are exhibited by the emission ratio of the fluorophores (i.e. FRET ratio)^25^.

### *In vitro* characterization of FLIP-G

**Figure 3:**
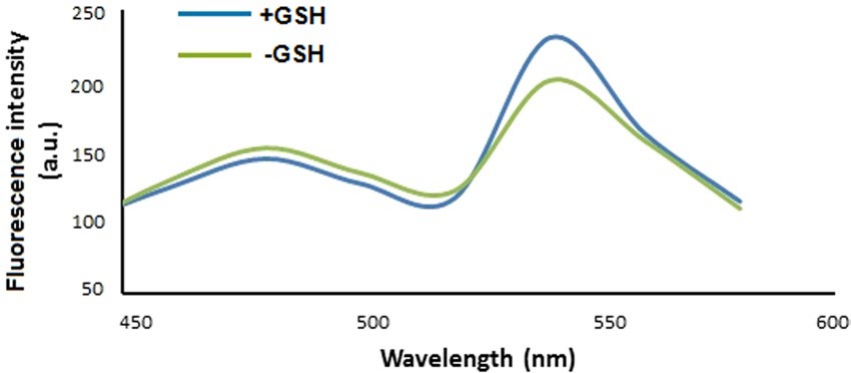
Fluorescence emission spectrum of GSH sensor. GSH (absence and presence)-based change in the emission spectra from purified FLIP-G protein was recorded that clearly indicates the energy transfer from CFP to YFP after binding with GSH. Sensor protein concentration was kept at 0.20 mg/mL.

Purified sensor protein from *E*. *coli* was used for fluorescence *in vitro* assays. Emission spectrum of the sensor protein was recorded and it revealed that the GSH addition in protein solution results in the decrease in fluorescence intensity of donor FP (CFP) and increase in fluorescence intensity of acceptor FP (YFP). These results demonstrated that the binding of GSH with YliB brought conformational change in YliB, which was translated into increased fluorescence intensity of YFP due to the proximity of two fluorophores (Fig. 3). Conformational change in the ligand-binding domain has been a basic requirement for the construction of FRET-based nanosensors, and also for translating metabolite binding into a change in FRET^26^. The sensor (FLIP-G) developed in this study meets this criterion, as the addition of GSH causes enough change in the YFP/CFP emission ratio.

**Figure 4:**
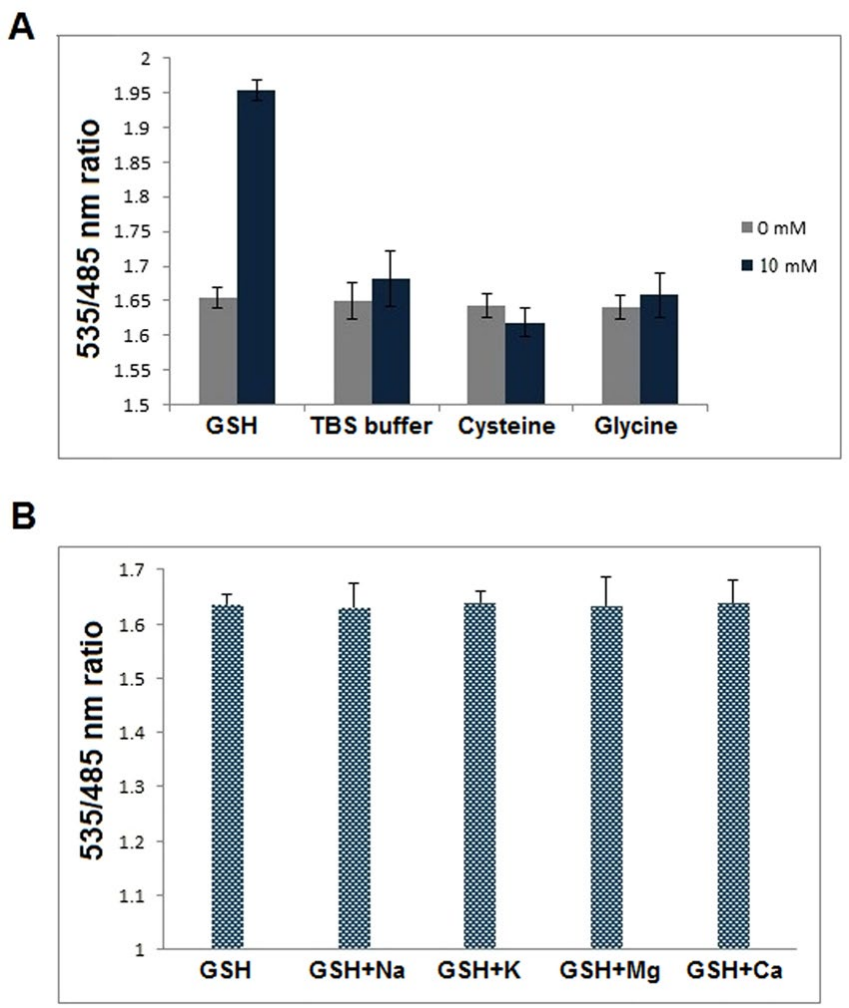
*In vitro* specificity analysis: (**A**) Specificity of the developed FLIP-G was checked in the presence of GSH and other compounds (TBS buffer, cysteine and glycine) at a concentration of 0 and 10 mM in microplate reader; (**B**) Metal specificity of FLIP-G was checked in the presence of different monovalent and divalent ions which do not interfere with the fluorescence of FLIP-G significantly. Sensor protein concentration was kept at 0.20 mg/mL. Excitation filter/slits (430/20 nm) and emission filters/slits for CFP (485/20 nm) and YFP (535/25 nm) were used. Values are means of three standard replicates (n = 3). Vertical bars represent standard errors.

**Figure 5:**
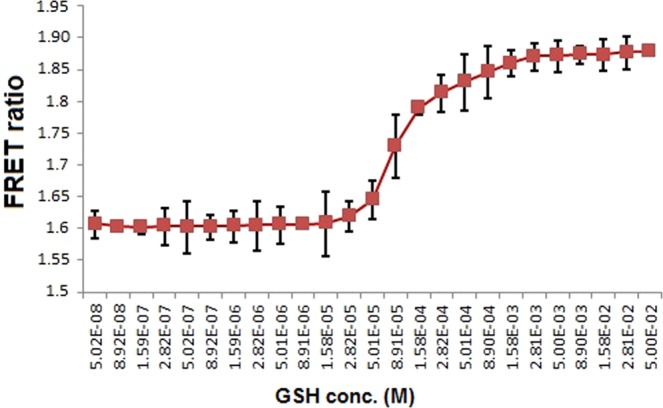
*In vitro* FRET measurement. Ligand-dependent changes in the YFP/CFP ratio of purified FLIP-G were recorded in the presence of GSH-concentrations. Ratio was increased by with increasing the concentration of GSH and was saturated at a particular concentration. Sensor protein concentration was kept at 0.20 mg/mL. Excitation filter/slits (430/20 nm) and emission filters/slits for CFP (485/20 nm) and YFP (535/25 nm) were used.

Fluorescence microplate reader with 96 well flat bottom plates was employed for i*n vitro* FRET assays. To assess the buffer suitability and stability of pH for FLIP-G, recording of emission intensity, and thereafter FRET ratio were calculated at different pH of various biological buffers. Among the various buffers tested, FLIP-G exhibited maximum stability and efficiency in TBS buffer (pH 7.2). Moreover, sensor protein was stabilized at physiological pH in TBS buffer (Fig. S3). Therefore, subsequent *in vitro* and *in vivo* analysis of the nanosensor was performed in TBS buffer with pH 7.2. The specificity of the developed nanosensor was also checked in the presence of different compounds. It was found that FLIP-G is very specific for GSH and did not show any significant changes in intensity ratio in the presence of other metabolites (Fig. 4A). Additionally, effect of metal ions on the fluorescence of sensor protein was examined in the presence of physiologically relevant metal ions. Metal ions such as Ca^2+^, Mg^2+^, Na^+^ and K^+^ did not cause any significant change in the fluorescence emission ratio (Fig. 4B). Titration analysis of FLIP-G with GSH concentrations from nM to mM resulted in a sigmoid curve and affinity constants (*K*_d_) was found to be ~282 µM (Fig. 5). Previously, the determination of the *K*_d_ for periplasmic binding proteins was done by equilibrium dialysis approach^27^. In our study, the affinity of sensor was calculated on the basis of FRET ratio change in the chimeric sensor protein at various concentrations of GSH. In fact, overlap between the donor emission spectrum and acceptor excitation spectrum, and proximity of donor and acceptor fluorophores are requisites for FRET to occur^27^.

### Live cell fluorescence measurement in *E*. *coli* cells

**Figure 6:**
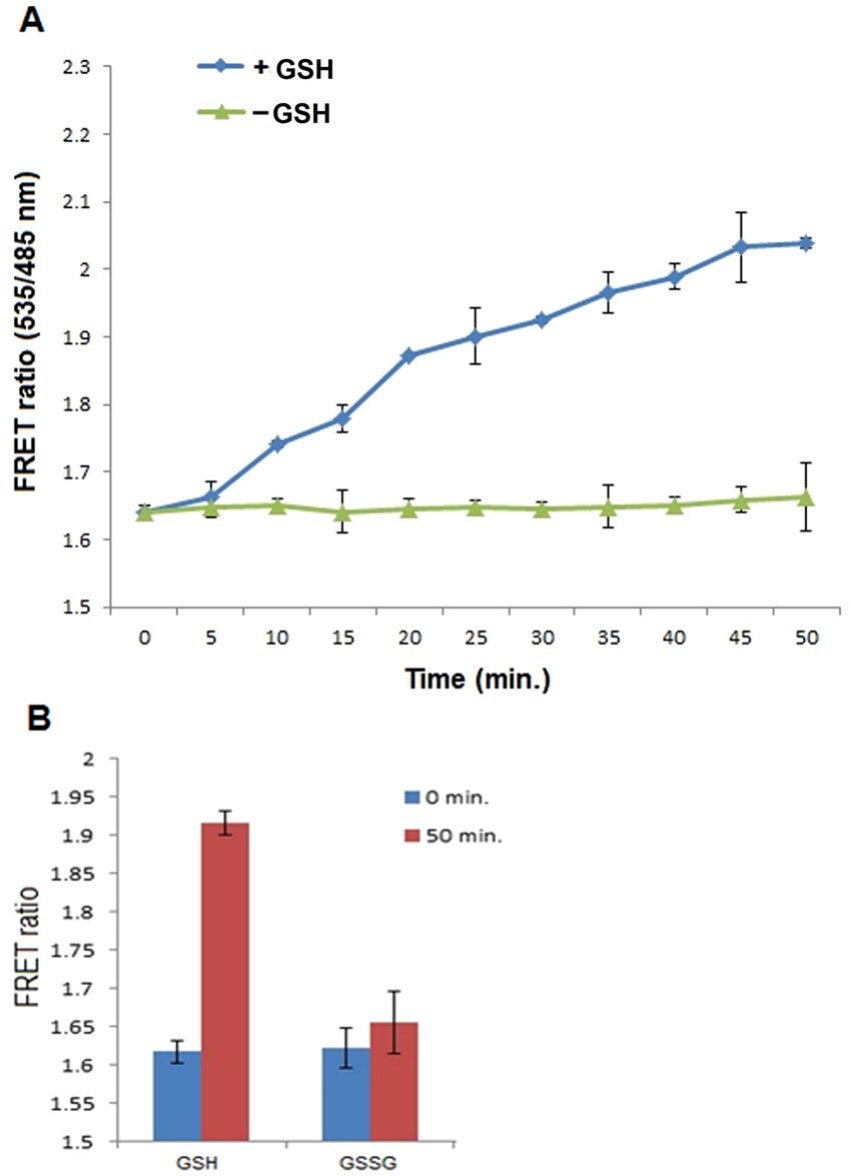
*In vivo* characterization in *E*. *coli* cells: (**A**) FRET ratio was recorded at defined intervals in the absence and presence of reduced glutathione (GSH). Ratio was found to increase with respect to time showing the cytosolic accumulation of GSH. Excitation filter/slits (430/20 nm) and emission filters/slits for CFP (485/20 nm) and YFP (535/25 nm) were used. (**B**) *In vivo* specificity of the sensor was monitored in *E*. *coli* cells and the GSH-dependent major change in ratio was observed. No significant change was observed in the presence of oxidized glutathione (GSSG).

The capability of FLIP-G for intracellular detection of GSH was first tested in live *E*. *coli* cells. Metabolite accumulation and measurement were as also done earlier in bacterial cells employing the FRET-based genetically encoded sensors^28,29^. In order to test the *in vivo* specificity and real-time monitoring of GSH, the sensor protein was allowed to express in *E*. *coli* cells. The harvested bacterial cells were suspended in TBS buffer. Suspension of these bacterial cells was supplemented with GSH in 96-well flat bottom plates. The resultant emission intensity of both fluorophores was recorded after a fixed intervals using fluorescence plate reader. Addition of GSH led to significant increase in the FRET ratio that reached to the saturation at 50 min of incubation (Fig. 6A). This elevation in the FRET ratio confirmed the ability of the developed sensor for monitoring the accumulation and alterations in the level of GSH that was being transported into *E*. *coli* cells. Additionally, there was no significant change in the intensity ratio observed in the presence of GSSG, which confirms the specificity of FLIP-G with reduced form (GSH) over the oxidized one (GSSG) *in vivo* (Fig. 6B).

### Ratiometric imaging of GSH in live yeast cells

**Figure 7:**
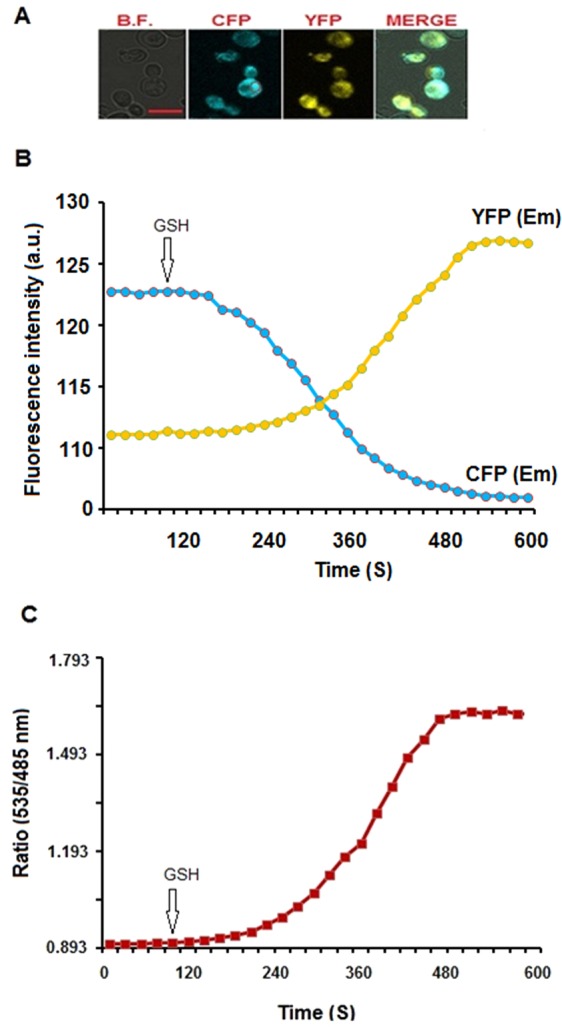
Real-time imaging of GSH changes in the cytosol of yeast cell: (**A**) Images of yeast cells expressing the FLIP-G were recorded by confocal microscope at different channels (i.e. bright field, CFP. YFP, and merge images; Scale bar- 5 µm); (**B**) Fluorescence emission intensity of CFP and YFP fluorophores was recorded over time in yeast cells at 28 °C; (**C**) Emission intensity ratio (535/485 nm ratio) of FLIP-G expressing yeast cells in the presence of 5.0 mM GSH (final concentration). External application of GSH causes the change in intensity ratio that denotes the import and binding of GSH with FLIP-G into the cytosol.

The sensor protein was allowed to express in bacterial cells and confocal images were acquired at respective channels by CLSM (Fig. S4). *In vivo* performance of the FLIP-G nanosensor was also evaluated in eukaryotic system. Live cell imaging of *S*. *cerevisiae*/ura3 strain BY4742 showed the expression therein of the sensor protein in the cytosol, where the cytosolic changes in GSH were detected (Fig. 7A). Intensity of both fluorophores was at basal level until 2 min. Hereafter, the addition of the GSH with the sensor expressing yeast cells showed a clear change in the fluorescence intensity of CFP and YFP (Fig. 7B). The FRET ratio was recorded and found increased from 0.893 to 1.653; and subsequently, the fluorescence intensity was saturated with time (Fig. 7C). It indicated that GSH was transported into the cytosol, where it was recognized by FLIP-G. The oxidized glutathione (GSSG) did not bring significant change in the emission intensity of CFP or YFP (Fig. S5). Earlier, the FLIPmal nanosensor monitored the changes in maltose in live yeast cells^26^. The authors reported the expression of FLIPmal in yeast cells, and measured the changes in the concentration of maltose by ratiometric imaging^26^.

## Materials and Methods

### Reagents

All reagents were purchased from Sigma Aldrich and used as received, unless otherwise stated.

### Computational studies and plasmid construction

Crystal structure of *E*. *coli* K-12 YliB in complex with GSH (PDB ID- 1UQW, resolution- 2.72 Å) was retrieved from RCSB protein data bank. Protein preparation wizard was used to prepare YliB; whereas, ligand was prepared employing LigPrep 2.6 workflow in Schrödinger suite. Bond orders were assigned and hydrogen bonds were added. Using the receptor grid generation function, docking grid was generated at the center of YliB with 2.0 Å size. Finally, Glide was used for docking analysis and different poses of GSH with YliB were taken. In order to develop FRET based sensor for GSH, YliB was used as GSH sensory domain and sequences of YliB gene were retrieved from Ecogene 3.0 database. Primers were designed, and synthesized from Integrated DNA technologies (IDT). Sequences of forward primer were 5′-GGA**AGATCT**TCCGCCAAAGATGTGGTGGTG-3′ with *Bgl*II restriction site and reverse primer were 5′-CG**CTCGAG**CGGTTGCAAATCCGCGTC TTCAAAG-3′ with *Xho*I restriction site (bold sequences represent the restriction sites). Genomic DNA of *E*. *coli* K-12 was used to amplify the YliB nucleotide sequences. CFP and YFP coding DNA was amplified by polymerase chain reaction from pDH18 vector (YRC, Washington, USA). A construct was prepared by joining the amplified gene fragments of CFP, YliB and YFP in pGEM®-T easy vector. The CFP-YliB-YFP fragment was excised from pGEM®-T easy and sub-cloned into pRSET-B vector (Invitrogen, USA) at *Eco*RI sites for the expression of sensor protein in bacteria (Fig. S1 in ESI), harboring the 6_His_ tag which helps in purification of the expressed protein. The CFP-YliB-YFP sequences were also shuttled into pENTR 4.0 dual selection vector to generate an entry clone. For expression in yeast, these sequences were then transferred to pYES-DEST52 vector (Invitrogen, USA) through Gateway cloning technology using LR clonase-II enzyme following the manufacturer’s instructions. *Sacharomyces cerevisiae*/ura3 strain BY4742 was used as eukaryotic host and transformed with pYES-DEST-CFP-YliB-YFP sequences. *S*. *cerevisiae* strain was maintained on Yeast Extract Peptone Dextrose (YEPD) agar medium and grown in liquid YEPD medium at 30 °C with aeration on a shaker.

### Sensor protein production and purification

The pRSET-CFP-YliB-YFP was transformed into *E*. *coli* BL21 (DE3) competent cells by electroporation method. The sensor protein was expressed as a fusion protein with an N-term 6_His_ tag and was purified employing nickel chelation affinity chromatography. A positive colony was grown in Luria broth (LB) medium supplemented with 100 mg L^−1^ ampicillin at 37 °C with shaking at 180 rpm till the optical density reaches to 0.6. Isopropyl β-D-1-thiogalactopyranoside (Fermentas, Germany) was added till a final concentration of 1.0 mM in order to induce the bacterial cells. The bacterial cells were grown at 20 °C and after 40–48 h, the cells were harvested by the centrifugation at 6500 × g for 10 min, washed 2–3 times with phosphate-buffered saline (PBS) buffer (pH 7.2) and the cell pellet was re-suspended in ice cold 20 mM Tris-Cl (pH 7.4). The cells were lysed in cell sonicator (Sonics, USA). Subsequently, the cell free extract was obtained by centrifugation at 6500хg for 30 minutes. Ni-NTA resin column was used to purify the GSH sensor protein (Novagen, USA). Cell free extract was mixed with the equivalent Ni-NTA resin and kept at 4 °C for 3 h to allow the binding of His-tagged sensor protein. This mixture was filled in the affinity column and washing of the column was done by 20 mM Tris-Cl and 20 mM imidazole (pH 7.4). Sensor protein was eluted with elution buffer containing 20 mM Tris-Cl and 250 mM imidazole (pH 7.4). Sensor protein was kept at 4 °C overnight for proper folding to its native conformation. Purified sensor protein was stored at −20 °C after addition of glycerol to a final concentration of 20% for further use.

### *In vitro* spectral analysis of the purified GSH sensor

Initially, various buffer systems have been tried to characterize the sensor. 3-(N-Morpholino) Propane Sulfonic acid) (MOPS), Phosphate Buffer Saline (PBS), and Tris-Buffer Saline (TBS) buffers (20 mM each) were used in this study. Stability of the sensor protein was further confirmed in TBS buffer (pH 5.5 to 8.0), with and without the addition of GSH. Sensor protein was diluted to a concentration of 0.25 mg mL^−1^ in TBS buffer to carry out the further analysis. The change in FRET ratio (*Em*YFP/*Em*CFP) with respect to different buffers and various pH was checked using fluorescence microplate reader (Beckman Coulter, USA). Emission intensity of the purified sensor protein diluted in 20 mM TBS buffer (pH 7.2) was investigated using Cary eclipse fluorescence spectrophotometer (Agilent, USA). To obtain the fluorescence spectrum, CFP was excited by 430 ± 20 nm light, and a continuous emission spectrum was monitored from 450–600 nm in the absence and presence of GSH.

Potential influence of metal ions (Ca^2+^, Mg^2+^, Na^+^ and K^+^) on the fluorescence intensity of the nanosensor was also examined. For this, an equal volume (1.0 mM each) from CaCl_2_, MgCl_2_, NaCl and KCl was prepared in 20 mM TBS buffer with 1.0 mM GSH. In each well, 180 µL of diluted nanosensor protein and 20 µL from each solution were taken. The excitation filter for CFP was 430/20 nm and emission filters for CFP and YFP were 485/20 nm and 535 nm/25 nm, respectively in microplate reader. The affinity of the purified nanosensor was also investigated using the different concentration of GSH. The affinity constant (*K*_d_) was determined by fitting the ligand titration curve in a single site-binding isotherm:

$${\rm{S}}=({\rm{r}}-{{\rm{R}}}_{{\rm{\min }}})/({{\rm{R}}}_{{\rm{\max }}}-{{\rm{R}}}_{{\rm{\min }}})={[{\rm{S}}]}_{{\rm{bound}}}/{[{\rm{P}}]}_{{\rm{total}}}={\rm{n}}[{\rm{S}}]/({{\rm{K}}}_{{\rm{d}}})+[{\rm{S}}]$$

where [S], substrate concentration; [S]_bound_, concentration of bound substrate; n, number of equal binding sites; [P]total, total concentration of binding protein; r, ratio; R_min_, minimum ratio in the absence of ligand; and R_max_, maximum ratio at saturation with ligand. Three independent protein extracts have been used for all FRET measurement purpose. FRET ratio was calculated by dividing the emission intensity of YFP by CFP.

### *In vivo* experiment in *E*. *coli*

FRET ratio changes in live *E*. *coli* cells were recorded using fluorescence microplate reader. *In vivo* FRET measurements were performed according to *Gruenwald et al*.^30^ and *Kaper et al*.^31^. The *E*. *coli* cells expressing the FLIP-G were grown in LB medium at 37 °C up to an optical density of 0.6. and then, induction of isopropyl β-D-1-thiogalactopyranoside (1.0 mM, final concentration) was given to the culture that was grown for additional 40–48 h at 20 °C. For appropriate maturation of fluorophores, cultures were stored for 16 h at 4 °C. Cells were starved for nitrogen in a carbon free M9 medium for 3 h at 37 °C, harvested by centrifugation and adjusted to the optical density of 3.0 in 20 mM TBS buffer (pH-7.2). Re-suspended cells (180 µL) were transferred to the each well of black clear bottom 96 well plate. Subsequently, 20 µL of GSH (final concentration of 5.0 mM) was added to the wells. The excitation filter and emission filters were set at the same setting as described in above section. FRET ratio (*Em*YFP/*Em*CFP) was monitored with specified intervals.

### Live cell confocal imaging of bacterial and yeast cells

The FLIP-G expressing *E*. *coli* BL21 (DE3) cells were prepared according to the method described above under *in vivo* measurement section. Live cell images were acquired by Confocal Laser Scanning Microscope (CLSM, Leica Microsystem, Germany). Yeast were transformed with pYES-DEST-CFP-YliB-YFP, and colonies were grown in the synthetic defined growth media until the optical density was reached between 0.5–0.6. Glucose with a final concentration of 2% was added to the medium as a carbon source; whereas in the same medium, galactose with a final concentration of 1% was added as a growth inducer. The colonies were let to grow for 2–3 days. Yeast cells were harvested and fixed on a poly L-lysine coated cover slide by using medical adhesive. GSH (5.0 mM final concentration) was added to the cells positioned on the glass slide. Live cell yeast imaging was performed by CLSM equipped with a confocal head TCS-SPE (Leica Microsystems,) and a 63x oil immersion objective. To measure the intracellular level of GSH in yeast, YFP to CFP emission intensity ratio was recorded with 435 nm excitation filter and two emissions filters (485 nm for CFP and 535 nm for YFP) using LAS-AF software. FRET sensitized emission tool of LAS-AF software after background subtractions was used to display the data. The region of interest was chosen to calculate the intensity ratio changes in real-time.

## Conclusions

Non-invasive and specific detection of antioxidant molecule is crucial as it can help to determine the status of oxidative stress as well as the progression of disease in living cells. The developed herein genetically encoded FRET-based sensor (FLIP-G) has a low detection limit and can measure GSH selectively. Cytosol targeted versions of the sensor was stably expressed in *E*. *coli* and yeast, and GSH concentrations were assessed at single cell level using confocal microscopy and ratiometric image analysis. FLIP-G sensor could overcome the delivery and toxicity problems of chemical dye-based probes for GSH, and can also be used for real-time cytosolic monitoring of GSH, non-invasively. Thus, FLIP-G can serve as novel tool for investigating the occurrence of oxidative stress and imaging the redox status in living cells. Because FLIP-G sensor is genetically encoded, it can be targeted to specific cellular compartments of a living cell, and can also successfully measure the GSH as many times as required.

## Supplementary information

Supplementary information.
