# Supplementary material for: Real-time monitoring of glutathione in living cells using genetically encoded FRET-based ratiometric nanosensor
Source: Sci Rep. 2020 Jan 22;10:992. doi: 10.1038/s41598-020-57654-y (PMC6976633; doi:10.1038/s41598-020-57654-y)
Supplement: Supplementary file 1 — Supplementary information. [file 41598_2020_57654_MOESM1_ESM.pdf]

Real-time monitoring of glutathione in living cells using genetically encoded FRET-based ratiometric nanosensor

Mohammad Ahmad1, Naser A. Anjum2, Ambreen Asif2, Altaf Ahmad2*

1Department of Botany, School of Chemical and Life Sciences, Jamia Hamdard, New Delhi, India
2Department of Botany, Faculty of Life Sciences, Aligarh Muslim University, Aligarh, India

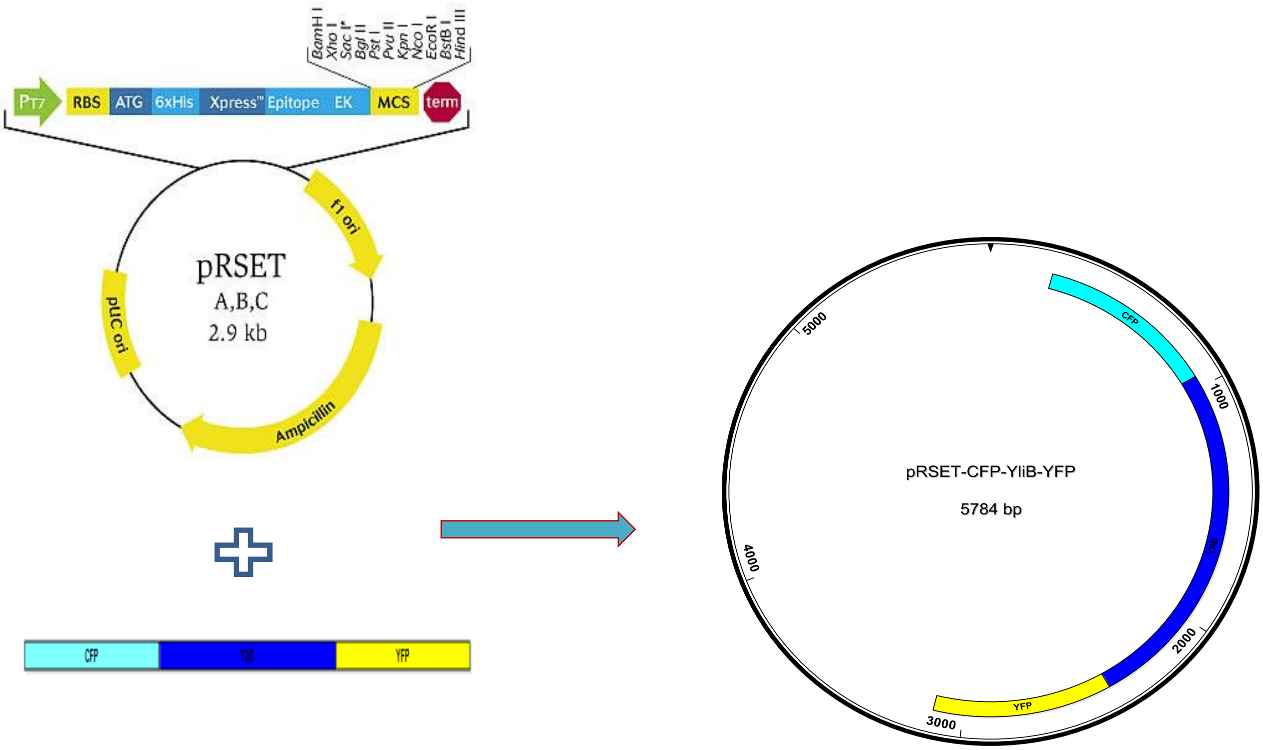

Fig. S1 Sub-cloning of the CFP-YliB-YFP construct in pRSET-B vector.

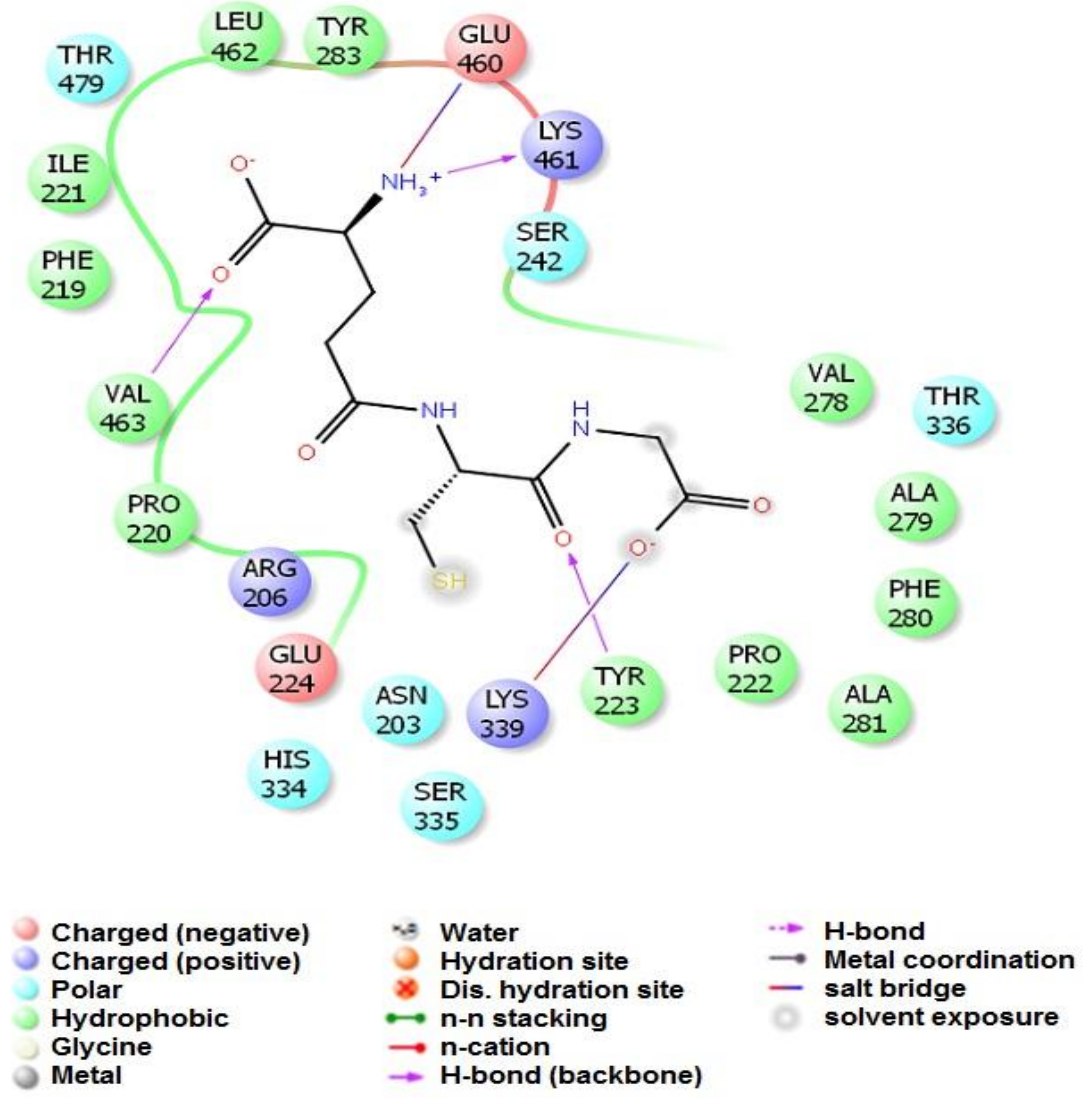

Fig. S2 GSH showing the various types of interaction at the active site. Hydrophobic interaction with Tyr283, Leu462, Ileu221, Phe219, Val463, Pro220, Tyr223, Pro222, Ala281, Phe280, Ala279, Val278

Polar interaction with Thr479, Ser242, His334, Asn203, Ser335, Thr336 and

Charged interaction with Glu460, Glu224, Arg206, Lys339, Lys461

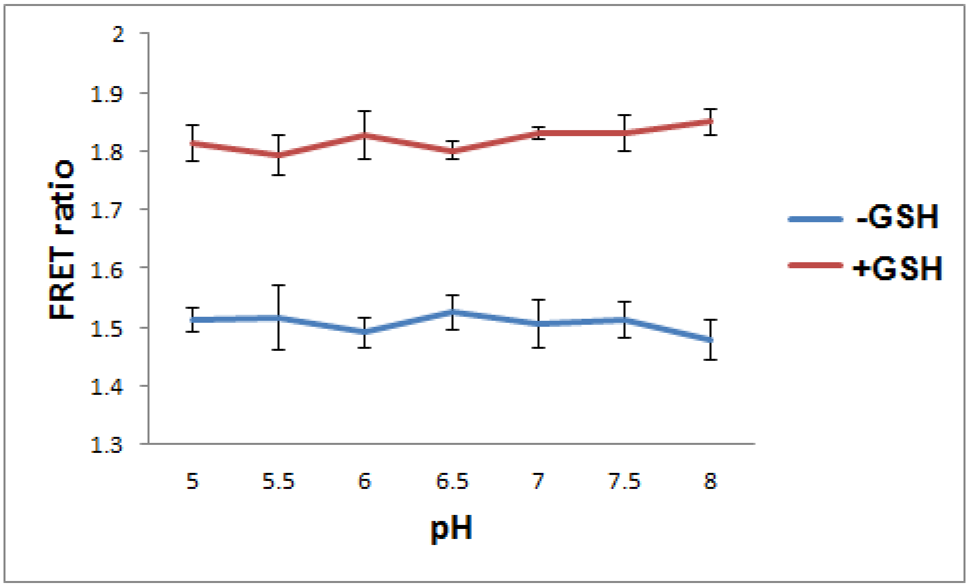

Fig. S3 pH stability of FLIP-G was tested in TBS buffer at different pH in the absence and presence of reduced glutathione (GSH).

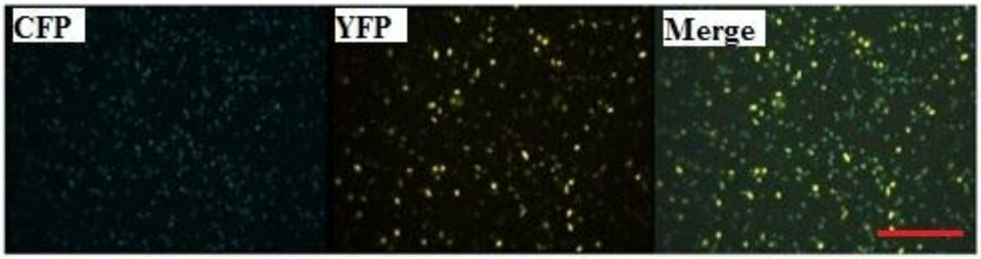

Fig. S4 *E.coli* cells were allowed to express the sensor protein and confocal images were recorded (scale bar- 5 µm). In this figure, images of bacterial cells were represented at CFP, YFP and merge channels.

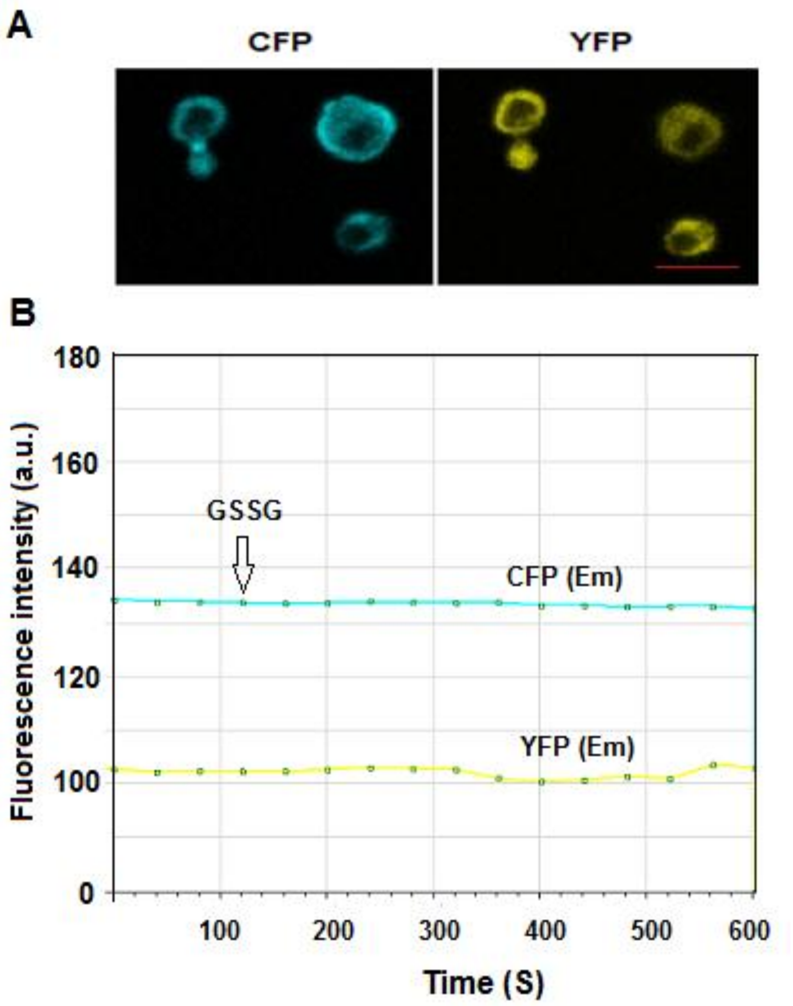

Fig. S5 Live cell imaging of yeast cell in the presence of oxidized glutathione (GSSG). Images of yeast cells expressing the FLIP-G were recorded by confocal microscope (Scale bar- 5 µm) (B) Fluorescence emission intensity of both the fluorophores was recorded over time in yeast cells in the presence of GSSG and it was found that addition of GSSG did not cause any significant change in the CFP and YFP fluorescence.
